# A Comparison Between Parents with Different Attitudes Towards Topical Fluoride Application for Their Children: A Cross-Sectional Study

**DOI:** 10.3290/j.ohpd.c_1804

**Published:** 2025-01-09

**Authors:** Hilal Özbey İpek, Arif Bolaca

**Affiliations:** a Hilal Özbey İpek Assistant Professor, Pamukkale University, Faculty of Dentistry, Department of Pediatric Dentistry, Kınıklı, Denizli, Turkey. Study concept and design, performed analysis, 1st author of the manuscript, read and approved the final manuscript.; b Arif Bolaca Assistant Professor, Pamukkale University, Faculty of Dentistry, Department of Pediatric Dentistry,Kınıklı, Denizli, Turkey. Study concept and design, administered surveys to the parents, performed analysis, co-wrote, read and approved the final manuscript.

**Keywords:** Keywords:

## Abstract

**Purpose:**

Although fluoride is known to be effective and safe, an increasing number of parents refuse to allow fluoride applications for their children. This study aimed to compare the parents who accepted and rejected fluoride application for their children in terms of their attitudes toward fluoride and vaccinations, sociodemographic characteristics, and source of knowledge.

**Materials and Methods:**

In this cross-sectional study, a previously validated questionnaire was administered to 85 parents who did not consent to have topical fluoride applied to their children’s teeth (AF group) and the 143 parents who consented to have it applied (F group) in a pediatric dentistry clinic. Data were analysed using the independent t-test and chi-squared test.

**Results:**

In the F group, the number of those who disagreed with the statement that fluoride causes intellectual disability, autism, and damages the pineal gland was statistically significantly higher than in the AF group (p < 0.05). The most common source of information for the AF group was the internet/social media (67.06%), while for the F group, it was dentists/medical doctors (62.24%). More parents in the F group stated that childhood and Covid-19 vaccinations must be performed; this was a statistically significant difference (p < 0.05).

**Conclusion:**

No relationship was found between fluoride hesitancy and the educational level of the parents. Most parents in the AF group have doubts and concerns about fluoride rather than being strongly opposed to it. Therefore, educational programs given to parents are likely to have a positive effect on their acquisition of correct information.

Fluoride has a powerful impact on the oral health of millions of adults and children. It is a unique member of the halogen group, in that it is termed a seeker of mineralised tissue. It is this affinity with mineralised tissues that explains how fluoride can strengthen and protect the teeth.^3,8^ The American Academy of Pediatric Dentistry (AAPD) recommends the use of fluoride for the prevention and control of caries, as it is highly effective in reducing caries prevalence. The AAPD recommends professionally-applied topical fluoride treatments at least twice per year to reduce caries incidence.^4^

Although fluoride is known to be effective and safe, an increasing number of parents refuse to allow fluoride applications for their children.^12^ This situation requires the attention of dentists and the scientific community. However, more parents are hesitant to use fluoride in their children than reject it in their children. This means that they may accept fluoride for their children but have concerns.^13^ In some studies, parents reported that they thought fluoride was toxic, that it would affect their children’s development and intelligence levels, and that they had concerns about the economic interests of pharmaceutical companies.^11,13,21,26^ Parents indicated that their primary concern was the desire to protect their children from potential harm.^11^

Science skepticism is a phenomenon that is growing around the world and influencing parents’ decisions. Similar to fluoride rejection and hesitancy, many doubt the benefits of modern medicine, including childhood vaccines and Covid-19 vaccines.^2^ Chi

## MATERIALS AND METHODS

### Study Design and Sample Size

This cross-sectional study was approved by the Ethics Committee of Pamukkale University, Faculty of Medicine (No.06; 2022/04) and all the procedures performed in the study were performed in accordance with the ethical standards given in the Declaration of Helsinki. Reporting was done in accordance with the Strengthening the Reporting of Observational Studies in Epidemiology (STROBE) statement.^29^ Parents of pediatric patients who applied to the Pamukkale University Faculty of Dentistry, Department of Pediatric Dentistry, and whose child had an indication for fluoride application were recruited from April 2022 to January 2024. Parents who accepted fluoride applications for their children and those who did not were compared in terms of their education levels, socioeconomic status, sociodemographic characteristics, perspectives on fluoride and vaccines.

The sample size was determined using G*Power 3.1. software (University of Kiel; Kiel, Germany) with 95% confidence (1-α), 95% test power (1-β), an effect size of d = 0.518, and mean of total knowledge as the primary outcome measure.^24^ It was calculated that a minimum sample size of 82 in each group was required.

The survey was terminated when the number of parents who did not consent to have topical fluoride applied to their children reached 85 and the number of parents who consented to have it applied reached 143. Data collection took approximately 22 months between April 2022 and January 2024.

### Validity of the Survey

The survey items were prepared based on other studies^6,13,20,24^ and were translated from English to Turkish. The content validity of the questionnaire was evaluated in pre-tests. To ensure the content validity, five expert dentists were asked to evaluate the items according to their relevance to the goals (items with high relevance = 1, moderate = 2, low or uncertain = 3). The content validity index ranged from 0.8 to 1. The items scored 2 or 3 were either deleted or modified accordingly.^24^

### Administration of the Survey

Informed consent was obtained for each participant and they were assured that their information would remain confidential. The exclusion criteria for the study were: not knowing enough Turkish to understand the survey questions and not consenting to fill out the survey. After the survey questions were explained to the participants in detail, they manually filled out the survey on their own. The survey had four sections. The first section started with sociodemographic data (age, occupation, educational level, total household income); the second section assessed the attitudes toward fluoride applications; the third section consisted of one item regarding sources of fluoride knowledge, and the final section focused on the parents’ perspectives on childhood vaccines and Covid-19 vaccines.

### Statistical Analysis

In this study, statistical analyses were performed with the NCSS (Number Cruncher Statistical System, 2007; Kaysville, UT, USA) package. In the evaluation of the data, in addition to descriptive statistical methods (frequency and percentage distributions, mean, standard deviation), the distribution of the variables was examined with the Shapiro-Wilk normality test. The independent t-test was used for pairwise compariston of groups of normally distributed variables, and the chi-squared test was used to compare qualitative data. The results were evaluated at a significance level of p < 0.05.

## RESULTS

A total of 287 parents were verbally asked to participate in the survey, 236 of whom agreed and gave consent (response rate: 82.2%). Eight parents who answered the survey questions incompletely were excluded from the study. Table 1 shows the sociodemographic data of parents and monthly household income. No statistically significant difference was observed in terms of the distribution of the number of children between the AF and F groups (p = 0.473). No statistically significant differences were observed between two groups in terms of the average age of the mother (p = 0.671), the average age of the father (p = 0.814), mother’s educational level (p = 0.196) and father’s educational level (p = 0.384). In terms of income, the distributions of 0-5.000TL (Turkish Lira) and 5.001-10.000TL in the F group were found to be statistically significantly higher (p = 0.0001) than the AF group (Table 1).

There was a statistically significantly higher number of parents in the F group who agreed that fluoride strengthens tooth surfaces and increases protection against tooth decay (p = 0.0001). For the F group, the number of those who disagreed with the statement that fluoride causes intellectual disability and autism, and damages the pineal gland was statistically significantly higher than the AF group (p = 0.0001). For the F group, the number of those who did not know that the “intake of excessive doses of fluoride into the body may cause disorders in bone and dental tissue” was statistically significantly higher (Table 2). Statistically signficantly more parents in the F group stated that their children used fluoridated toothpaste (p = 0.0001) (Table 3). In the F group, there was a statistically significantly higher (p = 0.0001) number of parents who said they let their children use fluoride-containing products because they think that fluoride applications are beneficial to their health than was the case in the AF group (Table 4).

Regarding answers to the question “Where did you get your information about fluoride?”, in the AF group, internet/social media (p = 0.0001), books (p = 0.0001), scientific publications (p = 0.026), neighbour/friend/relative (p = 0.043) responses were found to be statistically significantly higher than in the F group. The response “recommendations of dentists or medical doctors” was given statistically significantly more often (p = 0.001) in the F group than in the AF group (Table 5).

Statistically significantly more parents in the F group vs the AF group stated that childhood vaccines (p = 0.0001) and Covid-19 vaccines (p = 0.0001) must be done (Table 6).

## DISCUSSION

The motivation for conducting the present study was the negative atttitudes toward fluoride, which have been frequently shared in the media recently, as well as the presence of negative thoughts about topical fluoride among the parents of patients who attended our clinic. Recent studies report a prejudiced attitude towards fluoride all over the world. It is observed that parents resist fluoride applications due to concerns that it will negatively affect their children’s development and intelligence level. The number of parents who reject topical fluoride applications is increasing, and this is becoming an important public health problem.^13,26^ The aim of the study was to compare two groups of parents at our clinic who accepted and did not accept the application of fluoride in their children, in terms of their attitudes toward fluoride/vaccinations, educational levels, monthly income, sociodemographic characteristics and sources of knowledge.

Kalyoncu et al^21^ examined the reasons for the refusal of parents who rejectedllow the application of topical fluoride varnish in a public school. According to the results, 15.8% of the parents stated that they were not sufficiently informed about the application, 26.3% did not think that the application was carried out in a suitable environment, and 26.3% thought that fluoride was harmful. Another study reported that some parents thought that fluoride application was related to the economic interests of pharmaceutical companies.^11^ In the present study, the number of parents in the F group who let their children use fluoride-containing products because they think that fluoride applications are beneficial to their health was found to be statistically significantly higher than the AF group. However, 52.94% of the AF group answered “I’m not sure, I have doubts/concerns” to the question “What do you think about fluoride-containing toothpastes and fluoride applications?” (Table 4). This finding shows that more than half of the AF group had doubts/concerns about fluoride and but their attitude towards fluoride could be more positive if they were given correct information and education. Regardless of parents’ education, parents today are still not fully educated and informed about the management of their children’s oral hygiene.^9^ It is necessary to provide preventive training for children’s oral health, emphasising that oral health is crucial for general health.

With the advancement of technology, people have begun to widely use mass media, particularly television and the internet. Additionally, it is obvious that individuals are open to guidance and often believe the news/posts they read or watch without feeling the need to question them.^17^ In a study evaluating fluoride-related posts on social media, it was reported that 63% of the posts were anti-fluoride. This study also reported that a majority of the posts about fluoride contain conspiracy theories and focus on negative effects of fluoride.^7^ In addition, it is known that anti-fluoride posts of social media “influencers” with many followers can attract a vast following. In one study, it was observed that the most common sources of information about fluoride were the internet (35.1%) and dentists (35.4%), and to a lesser extent, neighbours and relatives (19.5%).^23^ In studies examining information sources, it was determined that the most frequent source of information used by parents was social media.^20,25^ In the present study, in parallel with previous studies, it was found that the most frequent source of information for the F group is dentists or medical doctors, but the major source of information for the AF group was the internet and social media.

Studies have shown a statistically significant relationship between the educational level of parents and their knowledge, and that highly educated people possess more accurate information about preventive treatments.^6,20^ However, some studies have reported that the opinion that fluoride is harmful increases with the increased education level.^13,26^ Additionally, other studies have shown no statistically significant relationship between parents’ knowledge and gender, age, and educational level.^18,25^ In the present study, no relationship was found between parental education level and acceptance or rejection of fluoride applications. This could be due to the impact of multiple factors on parental knowledge.

According to findings of the study by Wyne et al,^30^ age and gender were not correlated with knowledge; similarly Tahani et al^24^ found that gender and economic status were not correlated to knowledge. In the present study, no relationship was found between age and gender and the acceptance of fluoride applications. However, it was observed that the economic status of the F group was statistically significantly lower than that of the AF group (Table 1).

In their study, Alshehri and Kujan^3^ reported that approximately 30% of the participants stated that their children used toothpaste containing fluoride, and 54.3% were not aware of the fluoride content of the toothpaste. They also stated that 52.23% of the parents thought that fluoride toothpaste could prevent caries. However, approximately 60% of parents disagreed with the statement that fluoride toothpaste can strengthen the resistance of tooth surfaces. In the present study, it was observed that the proportion of parents in the F group whose children used fluoride-containing toothpaste was statistically significantly higher. However, it is noteworthy that approximately half of the participants in both groups had no knowledge about the fluoride content of toothpaste (Table 3). This finding shows the importance of increasing parents’ awareness by informing them about this matter. In the F group, the opinions that fluoride strengthens tooth surfaces, increases protection against caries, does not cause intellectual disability and autism in children, and does not harm the pineal gland by causing calcification were found to be statistically significantly higher (Table 2).

Carpiano and Chi^10^ stated that 51.5% of parents rejected fluoride and 27.7% rejected vaccines. In a study conducted by Chi,^12^ it was observed that 36.2% of parents who rejected a vaccine also rejected fluoride application. In a similar study, it was reported that parents who have negative thoughts about vaccination in children, also have negative thoughts about fluoride applications.^13^ In the present study, statistically significantly more parents in the F group stated that childhood vaccines and Covid-19 vaccines “must be done”. However, 4.71% of the AF group stated that childhood vaccines are harmful to health and 20.18% stated they have doubts or concerns. Regarding Covid-19 vaccines, 23.53% of AF group stated that these vaccines are harmful to health and 43.86% stated they have doubts or concerns (Table 6). These findings suggest hesitancy about the vaccines in the AF group, and if these parents are given correct information and education, their opinions could change. In the present study, vaccine rejection was less common than fluoride rejection. This may be because vaccine rejection can lead to irreversible complications or even death, while fluoride rejection is likely to lead to less critical health problems. Therefore, parents’ risk-benefit assessments may have been influenced by this perspective.

The present study’s finding that the economic status of the AF group was statistically significantly higher than the F group is questionable due to the economic fluctuations seen in Turkey. The present study started in April 2022 and was completed at the beginning of 2024. The majority of the F group completed the survey early in the study, while the majority of the AF group took longer to complete it. Therefore, the majority of the AF group completed the surveys towards the end of the study. For example, the net minimum wage in Turkey, which was 4.253TL at the beginning of the study, was 17.002TL by the end of the study.^27^ For this reason, the answers given by the survey participants to the question “What is your approximate total monthly household income?” were not consistent at the beginning and end of the study. This can be considered as a limitation of the study. The socioeconomic status of the two groups should be evaluated with a new survey study. To obtain a global view, studies with large sample sizes can be conducted. Another limitation of the study is that vaccine rejections were determined based on parental statements, so their accuracy was uncertain. However, topical fluoride rejections were determined by the authors in the clinic and were accurate. However, vaccine rejection by parents could have been determined from medical records on whether they had their children vaccinated or not. The present study represents the Turkish population from a single region and was conducted in a single hospital. This is another limitation of the study. Future studies can be planned to include children from different regions of the country. The results may vary in different regions and different cultures.

In this study, it appears that the majority of parents who did not consent to application of topical fluoride in their children have more concerns about fluoride, rather than a clear tendency to reject it. The instinct to protect their children lies at the basis of these opinions of mothers and fathers who refuse fluoride applications and vaccines. Although excessive fluoride can lead to weakening of the bones and teeth, sufficient amounts contribute to strengthening the teeth and preventing caries. Thus, it is important to increase the awareness of the benefits of fluoride and to educate the population.^3^ Dentists, physicians and governments should jointly organise educational programs so that parents can obtain safe and accurate information about the effects of fluoride and vaccines. Educational programs given to parents will likely have a positive impact on children’s health. In particular, considering the strong power of social media today, these educational programs should not only be carried out face-to-face but also through the media. In addition, it would be fruitful to provide training to dentists and physicians to explain the reasons for fluoride/vaccine opposition in society and provide effective methods of communicating with parents.

## CONCLUSION

In this study, no statistically significant difference was found between the age and education level in the AF and F groups. It was determined that the most common source of information for the AF group was the internet/social media, while for the F group, it was dentists/medical doctors. In the answers given by the participants of the AF group to the fluoride and vaccine questions, it was notable that many parents responded with “I don’t know” and “I have doubts/concerns”. The survey results showed that there is no relationship between fluoride hesitancy and the education level of the parents, and also that parents may be negatively affected by false health posts in the social media. Most parents in the AF group have doubts/concerns about fluoride rather than sharp opposition to it. Therefore, educational programs for parents are likely to have a positive effect on their acquisition of correct information.
